# Lateral sacral meningocele presenting as a gluteal mass: a case report

**DOI:** 10.1186/1752-1947-4-81

**Published:** 2010-03-05

**Authors:** Afsoun Seddighi, Amir Saied Seddighi

**Affiliations:** 1 Department of Neurosurgery, Rajaie Hospital, Padegan Street, Qazvin, Iran; 2 Department of Neurosurgery, Shohada Hospital, Tajrish Square, Tehran, Iran

## Abstract

**Introduction:**

Lateral meningocele is a very rare disorder. It has been reported in patients with neurofibromatosis or Marfan's syndrome. Previous reports have described lateral meningoceles in the thoracic or cervical region. Lateral meningocele in the sacral area was reported in the literature only once.

**Case presentation:**

We describe a 3.5-year-old Iranian girl who presented with a lateral gluteal mass. Neuroimaging and intra-operative evaluation showed that the mass was a lateral sacral meningocele with spinal communication through the iliac bone. We also present a review of the literature about this entity.

**Conclusions:**

Although lateral meningoceles especially in the sacral region are rare disorders, their possibility should always be considered in young patients presenting with a paravertebral or gluteal mass.

## Introduction

A meningocele is an outpouching of leptomeninges through a developmental defect in the dura. The arches of the vertebrae at one or more levels are involved with protruded meningeal sac covered with only a layer of skin[1].

Lateral meningoceles are extensions of the dura and arachnoid through an enlarged neural foramen. These often occur in the setting of Marfan syndrome or neurofibromatosis type 1 but may also be seen as isolated anomalies. Although they occur in the thoracic or sometimes in the cervical region, localization at the sacral spine is very infrequent [2].

## Case presentation

We present the case of a 3.5-year-old Iranian girl who was referred to us from pediatric surgery clinic because of a gluteal mass. Her parents informed us that the swelling of the right buttock of their daughter had been present since her birth. The size of the mass had been increasing gradually. They did not mention any bowel or bladder dysfunctions. A local physician had aspirated the cyst twice, 8 and 4 months prior to this presentation. Clear fluid had been aspirated each time, bur the mass would always reappear after several weeks. Unfortunately the results of the chemical analysis of the fluid were not available.

**Figure 1:**
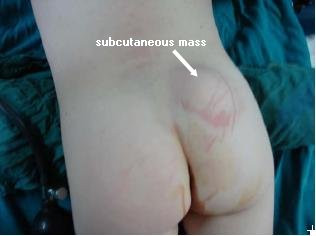
**Photograph of the patient showing the right lateral gluteal mass**.

A physical examination of our patient showed a soft, 3 × 4-cm fluctuant and non-pulsatile mass over her gluteal region. It was completely covered with normal skin without any vascular or hairy stigmata. The size of this mass did not increase with crying or coughing. Her transillumination test results were positive. Her midline was normal, with no sinus tract or swelling (Figure 1).

Results of her sensory, rectal tone and lower limbs motor exams were within normal limits. No abnormal skin lesions or skeletal deformities were found. The curvature of her spinal column appeared normal.

**Figure 2:**
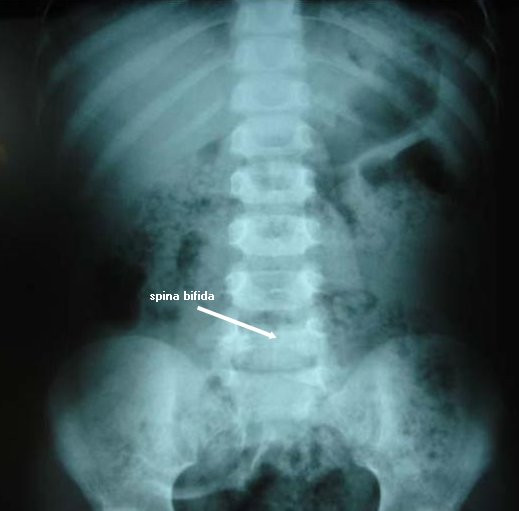
**X-ray, anteroposterior view of the lumbosacral spine showing L5 and S1 spina bifida**.

Results of her routine laboratory investigations were also normal. An anteroposterior X-ray examination of her lumbosacral region showed spina bifida of the L5 and S1 vertebrae (Figure 2).

**Figure 3:**
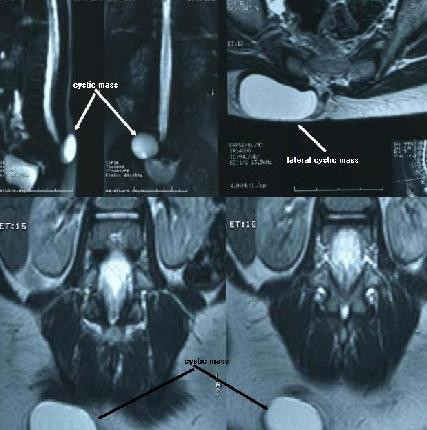
**Magnetic resonance image of the patient's lumbosacral spine**. The sagittal, axial and coronal view shows lateral sacral meningocele.

An ultrasound scan showed a very well-defined purely cystic, oblong lesion measuring 3.7 × 4.5 × 2.2 cm in size in our patient's right upper gluteal region under the gluteal muscles. There was no evidence of internal echoes or solid component or septae within the sac. We planned to perform myelography but unfortunately found it impossible because of our patient's allergic sensitivity to the contrast agent (omnipaque). A lumosacral magnetic resonance imaging (MRI) showed a well-defined cystic mass measuring 36 × 21 × 45 mm in her right buttock. The cystic mass appeared as a low signal intensity area on T1-weighted images and a high signal intensity area on T2-weighted images, which was similar to her cerebrospinal fluid (CSF) signal (Figure 3). No obvious communication between the cyst and the spinal canal was perceivable. The position and shape of the cord, roots and lying of the conus were unremarkable. The findings were suggestive of lateral meningocele.

A voiding cystourethrography showed a normal voiding pattern of our patient's bladder and urethra. There was no evidence of vesicoureteral reflux.

**Figure 4:**
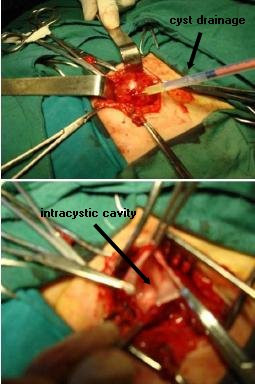
**An intra-operative view of the right gluteal lesion showing cystic mass containing cerebrospinal fluid**.

We then decided to perform resective surgery. After performing a transverse incision at the equator of our patient's lesion, we found a cystic, relatively thin-walled mass with a smooth pink exterior layer and a shiny creamy interior surface lying under the right gluteal muscles. We aspirated the cyst and clear fluid came out of it (Figure 4). Opening the cyst, we saw a very small aperture at the level of the ilium. We further extended the hole using microdrill and Kerrison punch along the tract, through which we saw communication between the cyst and the thecal sac through the spina bifida at the sacral canal. No neural tissue was found lying inside (Figure 4). After performing a ligature of the aperture of the cyst, we resected the walls and we closed the wound in layers. Our patient's postoperative recovery was uneventful.

**Figure 5:**
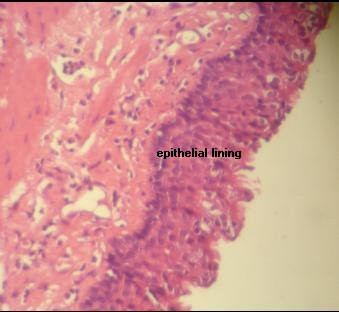
**Pathologic view of the resected mass showing fibrous tissue that has a lining of flattened to cuboid epithelium**.

On histopathology, we found that the wall of the sac was composed of fibrous tissue that had a lining of flattened to cuboidal epithelium. Polymorphonuclear leukocytes, histiocytes, and giant cells were also noted (Figure 5). The final pathological diagnosis was reported to be a meningocele with inflammatory reaction. Analysis of her intracystic fluid showed the following: red blood cells = 100/cc, white blood cells = 2500/cc (polymorphonuclear = 72%, lymphocyte = 28%), glucose = 60 mg/dl and protein = 45 mg/dl.

Our patient was found asymptomatic when she was presented for follow-up examination after 18 months.

## Discussion

Lateral meningoceles are considered as rare presentations of craniospinal dysraphisms [1,2]. These lesions were first described by Lehman in a patient with other skeletal findings and distinctive craniofacial features. He reported a 14-year-old girl with generalized osteosclerosis, distinctive craniofacial features, and multiple lateral thoracic meningoceles [3]. Subsequently, more patients with the so-called lateral meningocele syndrome (LMS) have been reported.

The existence of an affected mother and daughter supports the hypothesis that LMS is a dominant disorder affecting primarily the connective tissue [4]. Lateral meningoceles commonly present during the fourth and the fifth decades of life. Neurofibromatosis type 1 is present in approximately 85% of patients with lateral thoracic meningoceles. Meanwhile, the position of the cord with respect to the meningocele sac is variable.

The incidence of lateral meningoceles was reported to be 0.3% [4]. Lateral meningoceles are reported in the thoracic and lumbar regions followed by the cervical area [5-7]. Using various search engines such as Google, Pubmed, Alta vista, and a review of the literature, we found the entity of lateral sacral meningocele mentioned only once in the literature. It was presented by Navneet Kaur *et al*. in India [8].

Our patient did not show any evidence of abnormal pigmentation or skeletal deformities. The prominent feature of our patient is the isolated occurrence of the meningocele without any associated anomalies. The sac communicated with the lateral spinal canal only through a tract in her iliac bone.

Lateral meningoceles are usually associated with vertebral defects such as hemivertebrae, scoliosis, absence of neural arches on the affected side, and widening of the spinal canal and intervertebral foramina. Scalloping of the pedicles, laminae and vertebral bodies that are adjacent to the meningocele result in an enlarged spinal canal. Butterfly vertebra and segmental anomalies of the vertebral bodies may be found in as many as 43% of affected patients. Sacral anomalies, such as confluent sacral foramina and partial sacral agenesis, occur in up to 50% of reported cases [9,10].

In this case, the lumbosacral vertebrae showed normal appearance except for L5 and S1 spina bifida. Both our patient and the patient described by Navneet Kaur had spina bifida, which supports the presentation of sacral dysgenesis problems [8].

Lateral meningocele should be differentiated from other cystic sacral masses. It may be mistaken for a lipoma in a patient with lipomeningocele or for other cystic lesions such as cystic hygroma, synovial cysts, and large ovarian cysts [10]. Perineural or Tarlov cysts are asymptomatic and are discovered incidentally through myelogram or MRI originally intended for other reasons [11]. In diagnosing these cysts the contrast material does not readily enter the cyst during myelography and CT scan. Delayed filling is also typical, and MRI can be very useful in diagnosis [12].

In large ovarian cysts the determination of the origin of the mass can be difficult. These lesions can be demonstrated on computerized tomography scanning. They usually have a thin walls and attenuation values within the range of water. On MRI they exhibit low signal intensity on the T1- weighted sequences, high signal intensity on T2-weighted sequences, and are well-circumscribed with a thin wall that may enhance after contrast administration on T1-weighted images [13].

## Conclusion

Although lateral meningocele especially in the sacral region is rare, its possibility should always be considered in patients presenting with a paravertebral or gluteal mass. The occurrence of a neurological deficit or the presence of a spinal defect should make one suspicious of the presence of an unusually located meningocele. Drainage through needle aspiration or by incision may transform it into a cerebrospinal fluid fistula. Performing adequate imaging studies such as CT myelography and MRI, therefore, are very helpful to avoid mistakes and ensure correct diagnosis.

## Abbreviations

MRI: magnetic resonance imaging; CT: computerized tomography; RBC: red blood cell; WBC: white blood cell; PMN: polymorphonuclear; LMS: Lateral Meningocele Syndrome.

## Consent

Written informed consent was obtained from the patient's next-of-kin for publication of this case report and any accompanying images. A copy of the written consent is available for review by the Editor-in-Chief of this journal.

## Competing interests

The authors declare that they have no competing interests.

## Authors' contributions

AS analyzed and interpreted the patient data regarding the lateral sacral meningocele and performed the surgery. ASS aided in the surgery, performed the review of literature, and was a major contributor in writing the manuscript. Both authors read and approved the final manuscript.
